# Comparing protocols for preparation of DNA-free total yeast RNA suitable for RT-PCR

**DOI:** 10.1186/1471-2199-6-9

**Published:** 2005-04-15

**Authors:** Eduardo M Del Aguila, Marcio B Dutra, Joab T Silva, Vânia MF Paschoalin

**Affiliations:** 1 Departamento de Bioquímica, Instituto de Química, Universidade Federal do Rio de Janeiro, Centro de Tecnologia, Bloco A – Lab. 545 – CEP 21949-900 – Rio de Janeiro (RJ) – Brasil; 2 Centro de Ciências da Saúde, Universidade Salgado de Oliveira, Rua Lambari, 10- CEP 24456-570- São Gonçalo (RJ) – Brasil

## Abstract

**Background:**

Preparation of RNA free from DNA is a critical step before performing RT-PCR assay. Total RNA isolated from several sources, including those obtained from *Saccharomyces cerevisiae*, using routine methodologies are frequently contaminated with DNA, which can give rise to amplification products that mimic the amplicons expected from the RNA target.

**Results:**

We investigated the efficiency of two DNase I based protocols for eliminating DNA contaminations from RNA samples obtained from yeast cells. Both procedures are very efficient in eliminating DNA contamination from RNA samples and entail three main steps, which involve treating of RNA samples with DNase I, inhibition of the enzyme by EDTA and its subsequent inactivation at 65°C. The DNase I treated samples were further purified with phenol: chloroform followed by precipitation with ice-cold ethanol (protocol I) or, alternatively, they were directly used in RT-PCR reactions (protocol II). Transcripts from ACT1, PDA1, CNA1, CNA2, TPS1 and TPS2 analyzed after each treatment showed that all mRNAs tested can be amplified if total RNA was extracted and purified after DNase I treatment, however, only TPS1, TPS2 and ACT1 mRNAs were amplified without extraction/purification step.

**Conclusion:**

Although more laborious and requiring a higher initial amount of material, the inclusion of an extraction and purification step allows to prepare RNA samples that are free from DNA and from low molecular contaminants and can be applied to amplify any *Saccharomyces cerevisiae *mRNA by RT-PCR.

## Background

The adaptation of polymerase chain reaction (PCR) methodology to the investigation of RNA provides the researcher a method featuring speed, efficiency, specificity and sensitivity. Since RNA cannot serve as a template for DNA polymerase, a reverse transcription step was combined with PCR to transform RNA into a suitable complementary DNA (cDNA), a technique that is referred to as RT-PCR (reverse transcriptase – polymerase chain reaction) [1]. RT-PCR has enabled important experiments dealing with gene expression and its regulation. More sensitive and less laborious than Northern blotting hybridization and RNase protection assays, it has rapidly become a common procedure [2].

However, a frequent cause of concern among investigators performing quantitative RT-PCR is inaccurate data acquisition due to DNA contamination in RNA preparations, because PCR cannot discriminate between cDNA targets synthesized by reverse transcription and genomic DNA contamination. DNA contamination in RNA preparations is easily detected by performing a non-reverse transcriptase control. Furthermore, PCR primers can be designed for controlling the genomic DNA contamination. Primers that span intron-exon boundaries amplify a product from contaminating DNA that includes the intron, making it larger than the expected cDNA product. Alternatively, primers can be designed to anneal at a splice junction avoiding any signal based on DNA contamination [3]. Unfortunately, the genome of low eukaryotic or prokaryotic cells has few intron containing genes, which makes the above strategies useless. We have been faced with the problem of DNA contamination in our samples of RNA after initial attempts to study mRNA level in *Sacchromyces cerevisiae *by RT-PCR. Common methods used to remove DNA from RNA samples include poly (A) mRNA purification by oligo (dT) chromatography [4], selective RNA precipitation with lithium chloride [5] and selective DNA extraction with acid phenol: chloroform [6]. Oligo (dT) chromatography is expensive and requires extensive manipulation whereas LiCl precipitation and acid phenol: chloroform extraction could not be effective, mainly in the amplification of rare transcripts when an increasing number of cycles or amount of template RNA has to be used. An alternative method employs treatment of RNA samples with DNase I followed by heat inactivation of the enzyme [7]. Optionally, heat denatured DNase is extracted and RNA precipitated in order to avoid the presence of some compounds that could interfere with RT-PCR assay. The present study describes the comparison of two protocols for preparing yeast RNA free from DNA suitable for RT-PCR analysis based on DNase I removal of genomic DNA.

Here, we analyze the use in RT-PCR assay of yeast RNA samples isolated from routine techniques and treated by DNase I, in order to remove the DNA contamination. An additional step of RNA extraction followed by precipitation with ethanol must be included in order to guarantee that the unfractionated RNA samples are suitable for the analysis of expression of any *Saccharomyces cerevisiae *gene.

We hope that the re-evaluation of the methods for preparation of samples for RT-PCR showed here will encourage the use of this up to now under appreciated methodology for analyzing the mRNA levels in *Saccharomyces cerevisiae*.

## Results and discussion

**Figure 1:**
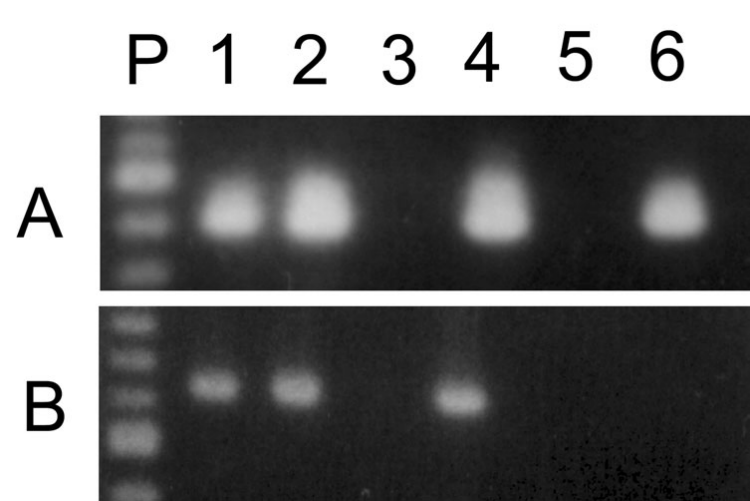
**DNA contamination in total RNA isolated from *Saccharomyces cerevisiae*. **Total RNA was isolated from *Saccharomyces cerevisiae *cells harvested at cellular density of 1.2 mg (dry weight/mL). PCR and RT-PCR amplicons were generated by 30 reactions cycles using *ACT1 *or *CNA1 *primers and 1.0 μg of RNA. Ten micro liters of each reaction was electrophoresed on a 1.3% agarose gel and stained with ethidium bromide. **A **– *ACT1 *amplicons. **B **– *CNA1 *amplicons. Untreated total RNA amplified by PCR (lane 1) or RT-PCR (lane 2); treated samples according to protocol I, followed by PCR (lane 3) or RT-PCR amplifications (lane 4); treated samples according to protocol II followed by PCR (lane 5) or RT-PCR amplification (lane 6).

**Figure 2:**
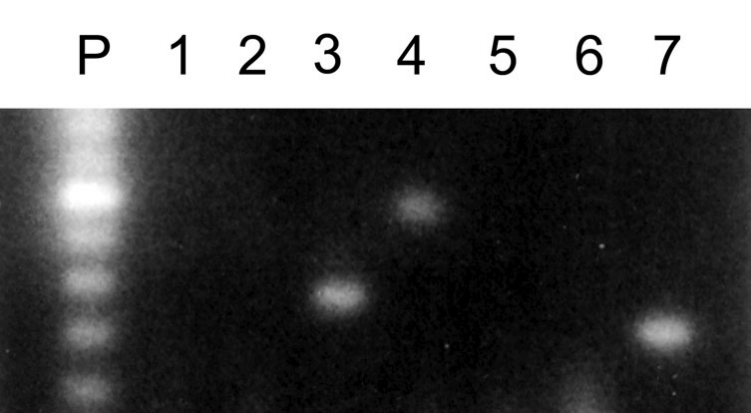
**Primer-template dependent inhibitory effect on mRNA amplification**. RNA samples extracted from cells harvested at 2,1 mg (dry weight)/mL were treated with DNase I according to protocol II. Different set of primers (as described in table I) was used to amplify *CNA2 *(lane 2), *ACT1 *(lane 3), *TPS2 *(lane 4), *PDA1 *(lane 5), *CNA1 *(lane 6), *TPS1 *(lane 7) mRNAs. 100 bp DNA ladder (lane P) and RT-minus control using ACT1 primers (lane 1).

**Figure 3:**
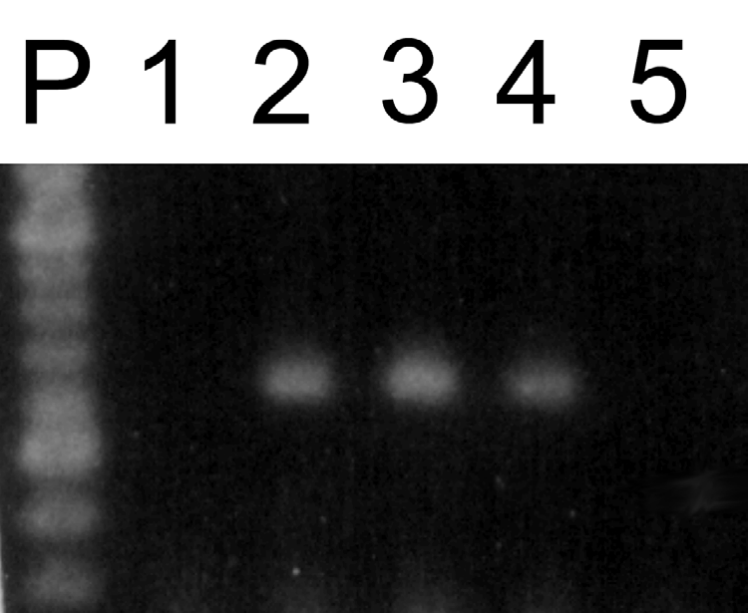
**Effect of EDTA concentration on *CNA1 *mRNA amplification by RT-PCR**. RNA samples extracted from cells harvested at 1.2 mg (dry weight/mL) were treated with DNase I according to protocol II. The reactions were stopped by the addition of 1.25 mM (lane 1), 1.5 mM (lane 2), 1.75 mM (lane 3); 2.0 mM (lane 4) and 2.25 mM (lane 5) of EDTA and treated samples were amplified by RT-PCR using *CNA1 *primers. P, 100 bp DNA Ladder (BioLabs-Inc).

Figure 1 shows the results of RT-PCR experiments using DNase-treated or untreated total RNA samples amplified using *ACT1 *(fig 1A) or *CNA1 *primers (fig. 1B). Untreated RNA samples produced the expected 407 bp (*ACT1*) or 629 bp (*CNA1*) amplicons, respectively, in the presence of active (fig. 1A and 1B, lanes 2) or heat inactivated reverse transcriptase (fig. 1A and 1B, lanes 1). Since RNA cannot serve as a template for DNA polymerase, these results make evident that DNA was present as a contaminant in the crude RNA preparations. Treatment of RNA samples with DNase I followed by RNA precipitation eliminated DNA contamination as judged by RT-minus control for *ACT1 *(fig. 1A, lane 3) and *CNA1 *reactions (fig 1B, lane 3). At the same time, it did not interfere with the production of *ACT1 *or *CNA1 *amplicons (fig. 1A and 1B, lanes 4) from their respective mRNA. Similar results were found for *ACT1 *reactions when DNase I treated samples were used without following precipitation (protocol II) (fig. 1A, lanes 5 and 6). On the other hand, *CNA1 *amplicons were not detected in RT-minus control neither in RT-PCR reactions using these same RNA-treated samples as template (fig. 1B, lanes 5 and 6). Based on these results, it seems that the use of treated RNA samples without the subsequent extraction/purification step produced an inhibitory effect on RT-PCR. To further address this question, we used a RNA sample treated according to protocol II as the source of templates to amplify six different mRNAs. *ACT1*, *TPS1 *and *TPS2 *transcripts generated the expected amplicons (fig. 2, lanes 3, 4 and 7), whereas *CNA1*, *PDA1 *and *CNA2 *failed to be amplified by RT-PCR (fig. 2, lanes 2, 5 and 6). In contrast, all of the six transcripts have been successfully amplified by RT-PCR if RNA templates were treated with DNase I followed by extraction/precipitation step with ethanol [8,9]. The compound that interfered on RT-PCR reaction could be EDTA, which is added to DNase I reaction in order to inactivate the enzyme. When carried over to the RT-PCR assay, EDTA could affect the free Mg^2+ ^concentration in the reaction mixture, changing the efficiency of amplification in a primer-template dependent way. To test if the excess of free EDTA carried over from DNase I treatment, indeed affects the efficiency of RT-PCR assay, DNase I activity was stopped by the addition of increasing amounts of EDTA (ranging from 1.25 to 2.25 mM), before the heat inactivation of the enzyme. The 629 bp *CNA1 *amplicons were detected when the reactions were stopped with 1.5 to 2.0 mM EDTA (resulting in a molar ratio from 0.7 to 1.2 of EDTA molecule to Mg^2+ ^ions (Fig. 3, lanes 2, 3 and 4). The addition of EDTA in concentrations out of this range abolished the amplification (Fig. 3, lanes 1 and 5).

**Figure 4:**
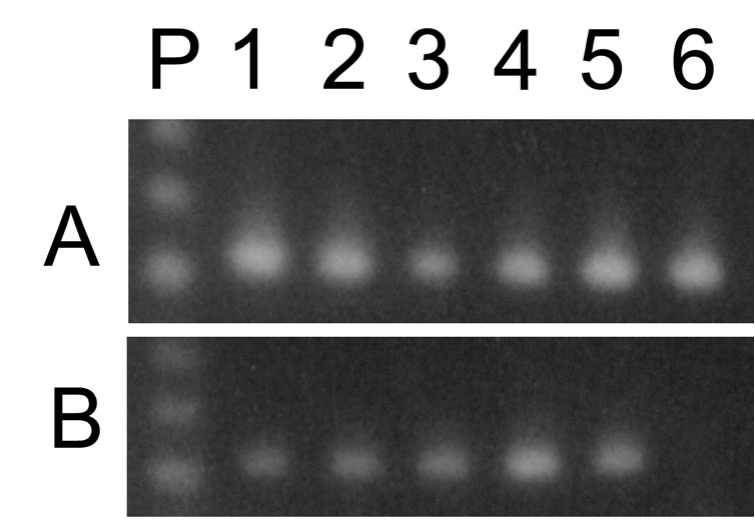
**Abundance of *ACT1 *transcripts during growth of Saccharomyces cerevisiae in glucose**. RT-PCR amplicons were generated by 30 reactions cycles using *ACT1 *primers and 1.0 μg of RNA. Ten micro liters of each reaction were electrophoresed on a 1.3% agarose gel and stained with EtBr. Total RNA was isolated from *Saccharomyces cerevisiae *cells grown in YPD-supplemented medium and harvested at 0.7 mg (dry weight)/ml (lane 1); 1.2 mg (dry weight)/ml (lane 2); 2.1 mg (dry weight)/ml (lane 3); 2.6 mg (dry weight)/ml (lane 4); 3.2 mg (dry weight)/ml (lane 5) and 7.0 mg (dry weight)/ml (lane 6). P, 123 bp DNA Ladder (BioLabs -Inc). **A **– Untreated RNA; **B **– RNA treated with Danes I by protocol I

To emphasize the fact that a simple DNase I treatment on crude RNA preparation is sufficient to perform accurate estimative of mRNA level by RT-PCR, we carried out the study of the expression of *ACT1 *mRNA along of the growth of an yeast strain in YPAD medium, using untreated and treated RNA samples obtained from cells harvested at different growth stages (Fig. 4, A and 4B). The amplification by RT-PCR using *ACT1 *primers and DNase I treated samples produced less intense bands (Fig 4B) at all growth phases when compared to those where untreated RNA was used (Fig. 4A). At stationary phase, using total RNA devoid of DNA contamination (Fig. 4B, lane 6) no expression of *ACT1 *genes was observed, a result consistent with those obtained using northern-blotting analysis [10], that showed a 100-fold decrease in *ACT1 *mRNA level at stationary phase.

In conclusion, total yeast RNA preparations can be made suitable for RT-PCR analysis of gene expression if they are free of contaminant DNA. DNase I treatment was effective in reducing to an undetectable level the DNA originally present in RNA samples. Different DNase brands can be used for this purpose. Here, DNase I preparations supplied form different manufacturers were used and both were very efficient in eliminating contaminant DNA. An additional step of extraction of reminiscent RNA with phenol: chloroform: isoamyl alcohol and precipitation with ethanol, as described in Protocol I, although more laborious, can be applied to amplify any *Saccharomyces cerevisiae *RNA using RT-PCR methodology. The main problem with this procedure is the need of starting with a higher amount of RNA to minimize loss of the sample during the precipitation step. Protocol II, on the other hand, is simple, practical and rapid because it avoids the extraction/precipitation steps and allows the direct use of treated RNA samples for RT-PCR assays. However, as magnesium concentration is a critical parameter in both RT and PCR reactions, the amount of EDTA used for inhibiting DNase I activity must be carefully titrated.

## Conclusion

RT-PCR can be a method for determining transcript level in total unfractionated yeast RNA, since the RNA samples are free from contaminant DNA.

DNA contamination from RNA samples can be efficiently eliminated by treatment with commercial DNase I preparations.

However, although simple and efficient, that treatment introduces chelant cation in RNA samples which makes it, in some cases, not suitable to be utilized in a subsequent enzymatic analysis like RT-PCR, because the enzymes used in that methodology, reverse transcriptase and DNA polimerase are Mg^2+^-dependent.

To ensure that no false positive result, or on the contrary, failure on mRNA amplification occur, we established a general procedure for any *S. cerevisiae *mRNA amplification, where an additional step of RNA extraction followed by precipitation with ethanol was included.

The re-evaluation of these methods described here will permit, a large scale or repetitive gene expression evaluation as those commonly performed during yeast utilization in industry, can be performed by RT-PCR, without additional cost, since even unfractionated RNA can be suitable for this methodology.

## Methods

### Yeast strain, growth conditions and extraction of total RNA

Total yeast RNA was isolated by a modification of the procedure described previously [11]. Strain W303-1A (*Mata, ade2-1*, *trp1-1*, *leu2,3-112*, *his3-11,15*, *ura3*, *can1-100*) was grown in YPD-supplemented medium (1% yeast extract, 2% bacto peptone, 2% glucose and 0,01% of adenine, uracil, tryptophan, leucine and histidine) in a rotatory shaker (160 rpm and 28°C). Cell growth was monitored by reading OD at 570 nm. Cells (10 mg of dry weight) were harvested by centrifugation and washed with DEPC (diethylpyrocarbonate) treated water at 2200 × g for 5 min before they were resuspended in 0.6 ml RNA extraction buffer (10 mM EDTA (ethylenediaminetetracetic acid), 50 mM Tris-HCl pH 7.5, 0.1M NaCl, 5% SDS) and 0.6 ml of phenol:chloroform:isoamyl alcohol (50:50:1) mixture. After 6 min at room temperature, 2 g of glass beads (0.45 mm diameter) were added and the cells were broken by vigorous agitation for 2 min on a vortex mix set at maximum speed. The extract was transferred to a microfuge tube (1.5 ml) and cell debris and organic phase were separated from upper aqueous phase by centrifugation at 2200 × g for 5 min (24°C). The upper phase was collected, extracted twice with 1 volume of phenol: chloroform: isoamyl alcohol (50:50:1) and once with 1 volume of chloroform: isoamyl alcohol (24:1). The RNA was precipitated from the last upper aqueous phase by the addition of 0.1 volume of 3M NaOAc, pH 5.2, plus 3 volumes of ice-cold absolute ethanol followed by incubation at -20°C for 1 h. The RNA was pelleted by centrifugation at 15000 × g for 15 min (4°C), washed once with ice-cold 70% ethanol and again pelleted at 15000 × g for 15 min. After the remaining alcohol was allowed to evaporate, the pellet was resuspended in 30 μl of DEPC treated water. Concentration of RNA in the sample was measured by reading OD at 260 nm in a Beckman DU-6 spectrophotometer (1 OD = 42 μg RNA/ml). All materials and solutions were previously treated with DEPC [12].

### Removal of contaminating genomic DNA from unfractionated RNA

RNA samples were treated with RNase-free bovine pancreatic DNase I (E.C. 3.1.21.1) to eliminate DNA contamination using two different protocols. Protocol I was a modification of the procedure previously described for *C. Botulinum *[13]. Six micrograms of RNA, 6.25 mM MgCl_2 _and 10U of RNase-free DNase I (Sigma) in a 10 μl reaction mixture in water were incubated at 37°C for 30 min. The reaction was stopped by the addition of 2 mM EDTA, pH 8.0, followed by incubation at 37°C for 1 min before inactivation at 65°C for 10 min. RNA was extracted with 1 volume of phenol: chloroform (5:1) and after centrifugation at 2200 × g for 5 min, the RNA present in the upper aqueous phase was precipitated with ice-cold absolute ethanol and collected by centrifugation at 15000 × g for 15 min (4°C). The remaining alcohol was allowed to evaporate and the pellet was resuspended in 30 μl of DEPC treated water. Protocol II was performed using the DNase I amplification grade kit (Life Technologies, Inc.) following the recommended procedure. In brief, 1 μg of RNA was resuspended in 20 mM Tris-HCl pH 8.4; 2.0 mM MgCl_2_; 50 mM KCl and 1U of DNase I into a final volume of 10 μl. After incubation for 15 min at room temperature, DNase was inactivated by the addition of 2,2 mM EDTA, pH 8.0, and subsequent incubation at 65°C for 10 min. The product of this reaction was used directly for RT-PCR without further treatment.

### RT-PCR assay

mRNAs were amplified by using the kit Ready-to-Go RT-PCR Beads (Amershan Pharmacia Biotech Inc.) in a GeneAmp PCR System 2400 (Perkin Elmer Inc.). Each reaction contained ~2.0U of TaqDNA polimerase, 10 mM Tris-HCl pH 9.0, 60 mM KCl, 1.5 mM MgCl2, 200 μM of each dNTP, MMuLV reverse transcriptase, RNA guard ribonuclease inhibitor, stabilizer including RNase/DNase free BSA, 1 μg of total RNA and 20 pmol of the appropriate primers (Del Aguila et al, 2003; Souza, 2001) (Table 1) into a final volume of 50 μl. Synthesis of cDNA was performed at 50°C for 20 min and stopped by inactivation of reverse transcriptase at 95°C for 10 min. Amplification of cDNA by PCR was performed for 30 sec at 95°C; 30 sec at 65°C and 1 min at 72° C for 30 cycles, chosen after testing amplification from 20 to 40 cycles. RT minus controls was incubated at 95°C for 10 min to inactivate M-MuLV (Moloney murine leukemia virus) reverse transcriptase before cDNA synthesis and PCR steps. RT-PCR products were run on 1.3% agarose gel in 1x TAE (40 mM Tris-acetate and 1 mM EDTA) buffer, pH.8.0, and stained with aqueous ethidium bromide at a concentration of 0.5 μg/mL [12]. Data represent a typical result obtained from three different experiments.

## Abbreviations

DEPC, diethylpyrocarbonate; EDTA, ethylenediaminetetraacetic acid; EtBr, ethidium bromide; M-MulV-RT, moloney murineleukemia virus reverse transcriptase; RT-PCR, reverse transcriptase – polimerase chain reaction

## Authors' contributions

EM performed all the experimental procedures and was the primary of the manuscript. MD participated in the study design. JT participated in the study design, data analysis and drafted the manuscript. VP conceived of the study, and participated in its design and coordination. All authors read and approved the final manuscript.

## Figures and Tables

**Table 1:** Oligonucleotides used as primers in RT-PCR analysis

**Target mRNA**	**Primer Sequence**	**Amplicon Size (bp)**
***TPS1***	**Forward **CGCTAAGGCGCAACTGACCTCGTCT **Reverse **CGACGAGAATGCGTGGTTGGCATAC	379
***TPS2***	**Forward **CCCCCCAAACTATCAGATTGGAACAAC **Reverse **ACCCAGCTGCAGCTATTCCATCGGC	612
***PDA1***	**Forward **GGTCAGGAGGCCATTGCTGT **Reverse **GACCAGCAATTGGATCGTTCTTGG	673
***CNA1***	**Forward **CGAAAGACTTGAATTCTTCACGCATC **Reverse **GAATGATCTGCAGCAAGCATCG	629
***CNA2***	**Forward **CCTTATATCTGTTCCCGCCC **Reverse **GAGGAACCATGGTTTTGGAG	551
***ACT1***	**Forward **CCTACGTTGGTGATGAAGCT **Reverse **GTCAGTCAAATCTCTACCGG	407

CNA1, CNA2, PDA1 and ACT1 primer sequences were obtained from Del Aguila *et al*, 2003 and TPS1 and TPS2, from Souza, 2001.
